# Experimental Validation and Bioinformatics Analysis Elucidate the Role of MTDH‐Mediated PTEN Ubiquitination and Degradation in Podocyte Injury in Diabetic Kidney Disease

**DOI:** 10.1155/humu/8914266

**Published:** 2026-05-13

**Authors:** Zerong Zheng, Danping Tao, Wenting Wu, Hui Zhang, Lingyu Shen, Xiaoyang He, Xiaohong Zheng, Yihao Long, Jinzhu Yang, Xiaowen Chen, Fenfen Peng, Haibo Long, Congwei Luo

**Affiliations:** ^1^ Department of Nephrology, Zhujiang Hospital, Southern Medical University, Guangzhou, China, fimmu.com; ^2^ Department of Gerontology, Zhujiang Hospital, Southern Medical University, Guangzhou, China, fimmu.com; ^3^ Integrated Traditional Chinese and Western Medicine Department, The Third People′s Hospital of Shunde District (Beijiao Hospital), Foshan City, Guangdong Province, China

**Keywords:** diabetic nephropathy, MTDH, podocyte cytoskeletal remodeling, PTEN, ubiquitin-conjugating enzyme E2N

## Abstract

Diabetic nephropathy (DN) stands as a primary contributor to end‐stage renal disease. Podocyte injury is a key factor underlying proteinuria in DN. Metadherin (MTDH) participates in podocyte apoptosis and promotes renal tubular injury in DN. However, its role in podocyte damage and podocyte cytoskeleton remodeling requires further investigation. PTEN plays a crucial role in maintaining podocyte integrity; however, the mechanisms governing PTEN stability in DN remain poorly understood. This study is aimed at investigating the specific functional role of MTDH in PTEN regulation using db/db diabetic mice, human kidney biopsy samples from DN patients, and cultured mouse podocytes exposed to high glucose (HG). MTDH expression was markedly increased in DN kidneys and HG‐stimulated podocytes. Elevated MTDH resulted in decreased PTEN protein expression levels without altering PTEN mRNA expression, suggesting a posttranscriptional regulatory mechanism. Further assays demonstrated that MTDH promoted PTEN degradation through the ubiquitin–proteasome pathway. Through bioinformatics analysis of the GSE96804 dataset from the Gene Expression Omnibus (GEO) database, obtaining 13,289 differentially expressed genes and comparing them with the known ubiquitin ligase–encoding genes obtained from the Genecards database, we identified candidate hub genes involved in PTEN ubiquitination‐mediated degradation. RNA sequencing identified ubiquitin‐conjugating enzyme E2N (UBE2N) as a critical downstream mediator positively regulated by MTDH. Subsequent coimmunoprecipitation experiments confirmed direct interactions between PTEN and UBE2N, enhancing PTEN ubiquitination. Knockdown of UBE2N attenuated MTDH‐induced PTEN degradation and podocyte cytoskeletal remodeling. Collectively, our findings reveal a novel regulatory axis wherein MTDH accelerates PTEN ubiquitination and proteasomal degradation via UBE2N, contributing to podocyte injury in DN. Targeting MTDH‐driven PTEN ubiquitination degradation presents a promising therapeutic strategy to protect podocytes and mitigate diabetic kidney injury.

## 1. Introduction

Diabetic nephropathy (DN), a severe complication of diabetes mellitus [1], is characterized by progressive renal damage due to chronic hyperglycemia. It affects 30%~40% of diabetic patients and is a leading cause of end‐stage renal disease (ESRD) [2]. The pathogenesis of DN is multifaceted, involving oxidative stress, inflammation, and hemodynamic alterations [3]. Despite advances in therapeutic strategies aimed at controlling glomerular hyperfiltration, halting progression to ESRD remains a significant clinical challenge.

Podocytes are pivotal for preserving the structural integrity of the glomerular filtration barrier. These cells attach to the glomerular basement membrane (GBM) through their foot processes (FPs), which are essential for a highly selective kidney filtration [4]. These FPs are stabilized by an intricate actin cytoskeleton, which provides mechanical strength and flexibility to withstand high filtration pressures. Key regulatory structures, such as the slit diaphragm (SD) and focal adhesions, control the dynamics of actin filaments, thus influencing FP mobility and stability. Injury to podocytes disrupts these structures, leading to cytoskeletal rearrangements that manifest as FP retraction, widening, and effacement [5]. This remodeling compromises the filtration barrier, contributing to DN progression [6]. Podocyte‐specific proteins, including nephrin, Neph1, and podocin, are indispensable for preserving the actin cytoskeleton and the structural integrity of podocytes [7, 8]. Synaptopodin, another important protein, contributes to actin filament bundling and helps maintain the dynamic cytoskeletal architecture, preventing FP effacement [9]. HG conditions, typical in diabetes, induce significant changes in podocyte phenotype, including the loss of essential cytoskeleton proteins such as podocin and synaptopodin, and the disorganization of the actin cytoskeleton, leading to FP effacement and detachment from the basement membrane [6]. These changes impair integrity of the glomerular filtration barrier, resulting in proteinuria, highlighting the importance of cytoskeletal dynamics in podocyte injury and DN progression.

MTDH was initially identified as a human immunodeficiency virus (HIV) and tumor necrosis factor‐*α* (TNF‐*α*) inducible‐type gene [10]. MTDH is an oncogene upregulated in many cancers, including breast, liver, and prostate cancers, and is involved in cell proliferation, differentiation, migration, and apoptosis [11]. Studies have shown that MTDH can induce tumor mesenchymal marker proteins and promote cancer proliferation and metastasis by regulating cytoskeletal remodeling [12]. However, research on MTDH in kidney diseases has primarily focused on podocyte apoptosis and oxidative stress–induced proteinuria, with few studies exploring its effects on the podocyte cytoskeleton remodeling in DN [13, 14]. PTEN is a tumor‐suppressor gene with phosphatase activity [15]. PTEN has proven to be downregulated in podocytes challenged with HG, and the potential renal protection of overexpressed PTEN in podocytes was partly attributed to an improvement in autophagy and motility and the inhibition of apoptosis [16]. In the renal cell carcinoma study, it was found that suppressing the expression of MTDH can upregulate PTEN protein expression levels in Caki‐2 cells [17]. The study demonstrated that MTDH is coexpressed and physically interacts with PTEN in resistant cancer cells [18]. In addition, NEDD4 and other E3 ubiquitin–protein ligases were all reported to have physical interactions or predicted genetic interactions from the Biological General Repository for Interaction Datasets with MTDH and PTEN [19]. We speculate that MTDH may be involved in the progression of DN by promoting ubiquitination degradation of PTEN and thereby affecting podocyte cytoskeleton and motility. However, it is unclear whether MTDH can affect the progression of DN by regulating the ubiquitination degradation of PTEN.

In this study, we emphasized that podocyte‐derived MTDH was a key driver of podocyte cytoskeleton remodeling in DN. Further in‐depth studies revealed the regulatory role of MTDH in PTEN ubiquitination and degradation and its impact on podocyte cytoskeletal rearrangement, providing a comprehensive understanding of its contribution to the pathogenesis of DN.

## 2. Results

### 2.1. MTDH Induces Podocyte Cytoskeletal Remodeling in DN

**Figure 1:**
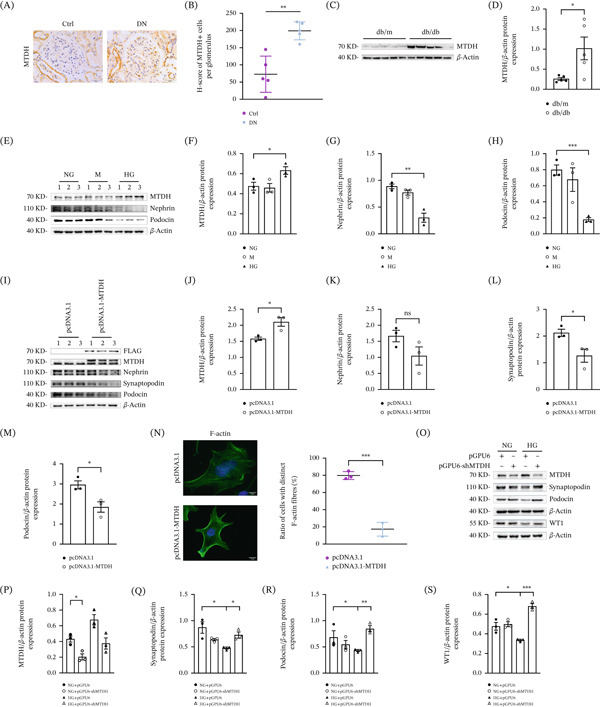
MTDH induces podocyte cytoskeletal remodeling in DN. (A, B) Representative micrographs illustrating renal MTDH expression across different groups. Paraffin‐embedded kidney sections were immunostained with antibodies against MTDH. Scale bar = 50 *μ*m. *H*‐score of MTDH staining in both groups,  ^∗∗^ *p* < 0.01 (*n* = 5). (C, D) Western blot analysis showing renal expression of MTDH in db/db and db/m mice. (C) Representative Western blot analysis results and (D) the corresponding quantitative data for MTDH are presented.  ^∗^ *p* < 0.05 versus db/m (*n* = 5). (E–H) Western blot analysis showing that HG intervention induced the protein levels of MTDH in vitro while simultaneously reducing the expression of podocin and nephrin. MPC5 cells were stimulated with 5.3‐mM glucose (NG group), 5.3 − mM glucose + 25 − mM mannitol (M group), and 30‐mM d‐glucose (HG group) for 48 h. (E) Representative Western blot results and (F–H) quantitative data on MTDH, nephrin, and podocin are provided.  ^∗^ *p* < 0.05,  ^∗∗^ *p* < 0.01,  ^∗∗∗^ *p* < 0.001 versus NG (*n* = 3). (I–M) Western blot analysis showing that overexpression of MTDH induced the protein levels of FLAG and MTDH while simultaneously reducing the expression of nephrin, synaptopodin, and podocin in vitro. MPC5 cells were transfected with control vector (pcDNA3.1) or MTDH overexpression plasmid (pcDNA3.1‐MTDH) for 24 h. (I) Representative Western blot and (J–M) quantitative data on MTDH, nephrin, synaptopodin, and podocin are presented. ns, no statistical difference;  ^∗^ *p* < 0.05,  ^∗∗^ *p* < 0.01,  ^∗∗∗^ *p* < 0.001 versus pcDNA3.1 group (*n* = 3). (N) Immunofluorescence staining of F‐actin to detect the effect of MTDH overexpression on the actin cytoskeleton in MPC5 cells. F‐actin was stained using phalloidin (green), and nuclear DNA was stained using DAPI (blue). Representative immunofluorescence images of F‐actin staining and quantification of F‐actin in podocytes transfected with different plasmids. Scale bar, 10 *μ*m.  ^∗∗∗^ *p* < 0.001 versus pcDNA3.1 group (*n* = 3). (O–S) (O) Representative Western blot and (P–S) quantitative data on MTDH, synaptopodin, podocin, and WT1 proteins from four groups are presented.  ^∗^ *p* < 0.05,  ^∗∗^ *p* < 0.01 (*n* = 3).

MTDH expression was elevated in both DN patients and db/db mice. Immunohistochemical staining revealed increased MTDH expression in glomerular podocytes of kidneys from DN patients (Figure 1A,B). Similarly, Western blot analysis showed that MTDH expression was significantly higher in kidney tissue proteins of db/db mice compared with db/m control mice (Figure 1C,D). Conditionally immortalized mouse podocytes (MPC5) were used for in vitro studies. To ensure a mature phenotype, cells were differentiated under nonpermissive conditions (37°C without IFN‐*γ*). Successful differentiation was confirmed by the marked induction of mature podocyte markers, including synaptopodin and nephrin, as assessed by Western blot analysis (Figure S1A). To establish the optimal experimental condition, MPC5 cells were exposed to a gradient of D‐glucose concentrations (e.g., 5.3‐mM normal glucose [NG], and 20, 30‐mM high glucose [HG]). We found that the expression changes of our target molecules were most pronounced at 30‐mM glucose (Figure S1B). Therefore, this concentration was selected for all subsequent high‐glucose treatment experiments. In HG‐treated MPC5 cells, WB experiments also detected elevated levels of MTDH and reduced podocyte cytoskeleton–associated proteins podocin and nephrin (Figure 1E–H).

We speculated that HG‐induced podocyte injury is associated with increased MTDH expression. To investigate this, we overexpressed MTDH in MPC5 cells using an MTDH encoding plasmid. Overexpression of MTDH resulted in a marked and significant decrease in the expression of synaptopodin, a FP cytoskeleton regulatory protein, as well as podocin, which are FP cell–specific proteins. This suggests that MTDH overexpression causes podocyte cytoskeletal remodeling (Figure 1I–M).

The podocyte cytoskeleton consists of actin and actin regulatory proteins. Actin exists in both monomeric (G‐actin) and multimeric (F‐actin) forms, with F‐actin playing a crucial biological role. The main actin regulatory proteins are *α*‐actinin‐4 and synaptopodin. Podocyte cytoskeletal remodeling involves changes in the polarity and tension of actin filament arrangement and altered phosphorylation levels of actin regulatory proteins. Immunofluorescence staining using phalloidin for F‐actin showed that, in normal MPC5 cells, F‐actin crosslinks were highly ordered and formed parallel bundles extending through the cell length and distributed in a polarized manner. Overexpression of MTDH led to podocyte cytoskeletal remodeling, with F‐actin disintegrating into a loose mesh structure, resulting in loss of cellular tension and cell body retraction (Figure 1N).

To further clarify the role of MTDH in HG‐induced podocyte injury, we utilized MTDH small hairpin RNA (shRNA) encoding plasmid to downregulate MTDH expression in MPC5 cells. WB results demonstrated that the loss of podocin, Wilms′ tumor Suppressor 1 (WT1), and synaptopodin caused by HG stimulation was significantly alleviated following the downregulation of MTDH, with all changes showing statistical significance (Figure 1O–S). These findings suggest that MTDH contributes to HG‐induced podocyte cytoskeletal remodeling and that downregulation of MTDH can mitigate HG‐induced podocyte injury.

### 2.2. MTDH Regulates PTEN‐Mediated Podocyte Cytoskeletal Remodeling

**Figure 2:**
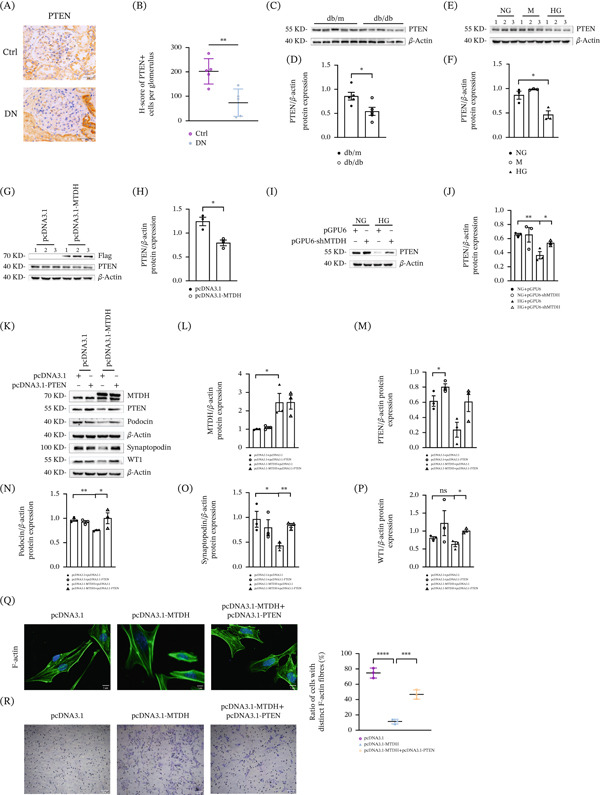
MTDH regulates PTEN‐mediated podocyte cytoskeletal remodeling. (A, B) Representative micrographs showing renal PTEN expression in different groups. Paraffin‐embedded kidney sections were immunostained with antibodies against PTEN. Scale bar, 50 *μ*m. *H*‐score of PTEN staining in both groups,  ^∗∗^ *p* < 0.01 (*n* = 5). (C, D) (C) Representative Western blot demonstrating renal PTEN protein expression in different groups, and (D) quantitative data on the relative abundance of renal PTEN proteins in different groups.  ^∗^ *p* < 0.05 versus db/m (*n* = 5). (E, F) Western blot analysis showing PTEN protein levels in MPC5 cells treated with NG, M, and HG for 48 h. (E) Representative Western blot results and (F) the quantitative analysis data on PTEN are presented.  ^∗^ *p* < 0.05 versus NG (*n* = 3). (G, H) Western blot analysis showing the effect of MTDH overexpression on PTEN protein levels in MPC5 cells. Cells were transfected with either the control vector (pcDNA3.1) or the MTDH overexpression plasmid (pcDNA3.1‐MTDH) for 24 h. (G) Representative Western blot and (H) quantitative data on PTEN are presented.  ^∗^ *p* < 0.05 versus pcDNA3.1 group (*n* = 3). (I, J) (I) Representative Western blot and (J) quantitative data on PTEN protein levels in the four groups.  ^∗^ *p* < 0.05,  ^∗∗^ *p* < 0.01 (*n* = 3). (K–P) (K) Representative Western blot and (L–P) quantitative data on MTDH, PTEN, podocin, synaptopodin, and WT1 protein levels in four groups. ns, no statistical difference;  ^∗^ *p* < 0.05,  ^∗∗^ *p* < 0.01 (*n* = 3). (Q) Representative immunofluorescence images of F‐actin staining and quantification of F‐actin in podocytes transfected with different plasmids. Scale bar, 5 *μ*m.  ^∗∗∗^ *p* < 0.001,  ^∗∗∗∗^ *p* < 0.0001 (*n* = 3). (R) The effect of MTDH or PTEN overexpression on MPC5 cell metastasis, assessed using the Transwell assay. Scale bar, 20 *μ*m.

PTEN expression was found to be reduced in both DN patients and db/db mice. Immunohistochemical staining revealed decreased PTEN expression in glomerular podocytes of kidneys from DN patients (Figure 2A,B). WB results confirmed that PTEN expression was diminished in renal tissues of db/db mice (Figure 2C,D). Similar results were observed in MPC5 cells treated with HG, where PTEN protein expression was significantly reduced (Figure 2E,F).

Notably, overexpression of MTDH resulted in a significant decrease in PTEN expression (Figure 2G,H). To further investigate the effect of MTDH on PTEN, we downregulated MTDH expression in HG‐treated MPC5 cells. WB results demonstrated that the inhibition of PTEN expression by HG intervention was alleviated after downregulation of MTDH, suggesting that suppressing MTDH expression can reverse the HG‐induced reduction of PTEN in MPC5 cells (Figure 2I,J).

To explore the role of PTEN in MTDH‐induced podocyte cytoskeletal remodeling, we used transient transfection to upregulate MTDH and PTEN expression in MPC5 cells. WB results showed that PTEN expression was significantly reduced in MPC5 cells overexpressing MTDH, accompanied by a significant reduction in the expression of podocyte‐specific proteins podocin, WT1, and the actin‐binding protein synaptopodin. However, the damage induced by MTDH overexpression was alleviated by PTEN overexpression, with all changes showing significant differences (Figure 2K–P).

Immunofluorescence staining of F‐actin revealed that overexpression of MTDH led to cytoskeletal remodeling and disorganization of actin filament arrangement in MPC5 cells, which were mitigated by PTEN overexpression (Figure 2Q). Furthermore, the Transwell migration assay indicated that MTDH overexpression increased MPC5 cell migration activity, whereas PTEN overexpression reduced this migration (Figure 2R). These results indicate that MTDH induces podocyte cytoskeletal remodeling by inhibiting PTEN protein expression under HG conditions.

### 2.3. MTDH Promotes Ubiquitin–Proteasome‐Mediated Degradation of PTEN

**Figure 3:**
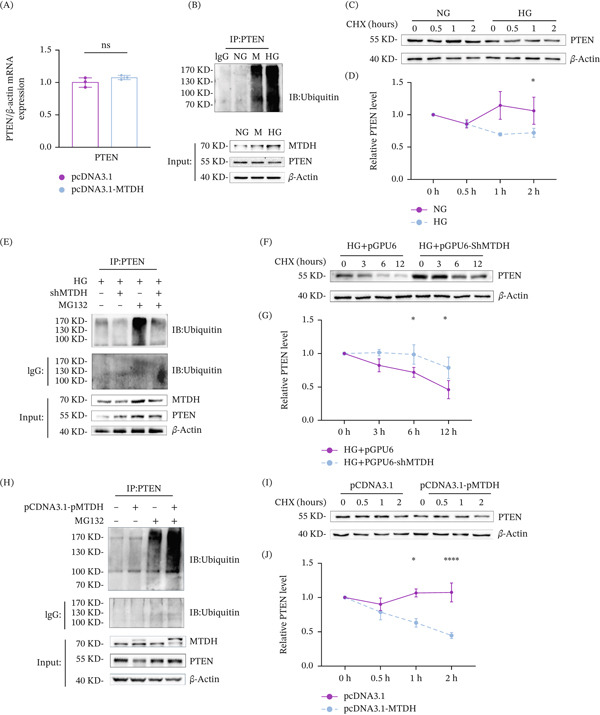
MTDH promotes ubiquitin–proteasome‐mediated degradation of PTEN. (A) QPCR results showing PTEN mRNA levels in indicated groups. ns, no statistical difference versus pcDNA3.1 group (*n* = 3). (B) Coimmunoprecipitation assays were performed to assess alterations in the ubiquitination level of PTEN. (C, D) (C) Western blot analysis results showing the levels of PTEN protein in the indicated groups. MPC5 cells were stimulated with NG or HG for 48 h, treated with CHX (40 *μ*M), collected at the indicated time points, and immunoblotted for PTEN and *β*‐actin. (D) Quantification of PTEN levels relative to *β*‐actin expression is shown.  ^∗^ *p* < 0.05 versus NG group (*n* = 3). (E) Coimmunoprecipitation assays were performed to assess alterations in the ubiquitination level of PTEN. (F, G) (F) Western blot analysis showing PTEN protein levels in indicated groups. MPC5 cells were transfected with pGPU6 or pGPU6‐shMTDH and cultured in HG medium for 48 h, then treated with CHX (40 *μ*M), collected at the indicated time points, and immunoblotted for PTEN and *β*‐actin. (G) Quantification of PTEN levels relative to *β*‐actin expression is shown.  ^∗^ *p* < 0.05,  ^∗∗^ *p* < 0.01 versus HG + pGPU6 group (*n* = 3). (H) Coimmunoprecipitation assays were performed in MPC5 cells transfected with pcDNA3.1 or pcDNA3.1‐MTDH to assess changes in the ubiquitination level of PTEN. (I, J) (I) Western blot analysis showing PTEN protein levels in indicated groups. MPC5 cells were transfected with pcDNA3.1 or pcDNA3.1‐MTDH for 24 h, then treated with CHX (40 *μ*M), collected at the indicated time points, and immunoblotted for PTEN and *β*‐actin. (J) Quantification of PTEN levels relative to *β*‐actin expression is shown.  ^∗^ *p* < 0.05,  ^∗∗∗∗^ *p* < 0.0001 versus pcDNA3.1 group.

We found that overexpression of MTDH had no effect on the mRNA levels of PTEN as assessed by qPCR (Figure 3A). Previous studies indicated that MTDH inhibits PTEN protein expression, suggesting that this regulation occurs at the posttranscriptional level, including posttranslational modifications such as acetylation, ubiquitination, and phosphorylation.

Western blot results showed that under HG conditions, the ubiquitination level of PTEN was significantly increased (Figure 3B). To further assess the stability of PTEN protein under HG conditions, MPC5 cells treated with HG were exposed to 40‐*μ*M cycloheximide (CHX) for 0 (control with DMSO), 0.5, 1, and 2 h. WB results indicated that HG conditions accelerated PTEN degradation (Figure 3C,D).

To investigate whether inhibiting MTDH could slow PTEN degradation under HG conditions, we administered specific shRNA to downregulate MTDH in MPC5 cells treated with HG. WB results showed that inhibition of MTDH expression significantly reduced the ubiquitination level of PTEN (Figure 3E). Additionally, cells were treated with 40‐*μ*M CHX for 0 (control with DMSO), 3, 6, and 12 h. WB results demonstrated that inhibiting MTDH expression under HG conditions significantly slowed PTEN degradation (Figure 3F,G).

To verify whether MTDH promotes PTEN degradation through the ubiquitin–proteasome pathway, we used a cell model that directly overexpressed MTDH. WB results showed that overexpression of MTDH in MPC5 cells resulted in reduced PTEN expression and a significant increase in PTEN ubiquitination (Figure 3H). Further assessment of PTEN stability in cells overexpressing MTDH treated with 40‐*μ*M CHX for 0 (control with DMSO), 0.5, 1, and 2 h revealed that overexpression of MTDH accelerated PTEN degradation, reducing its half‐life to as short as 1 h (Figure 3I,J). These findings suggest that MTDH promotes PTEN degradation via the ubiquitin–proteasome pathway.

### 2.4. Ubiquitin Ligase E3 NEDD4 Upregulated in Podocytes Exposed to HG

**Figure 4:**
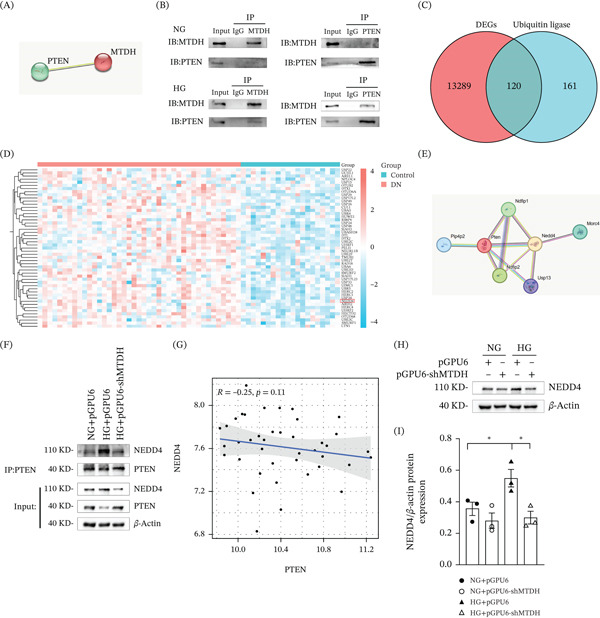
Ubiquitin ligase E3 NEDD4 upregulated in podocytes exposed to HG. (A) STRING database identified the interaction between MTDH and PTEN. (B) Interaction between PTEN and MTDH was confirmed through Co‐IP. MPC5 cells were stimulated with NG or HG for 48 h. mAb‐PTEN or mAb‐MTDH was used for immunoprecipitation, with rabbit IgG pAb as the control antibody. Western blotting was performed using mAb‐PTEN or mAb‐MTDH. (C) The Venn diagram of differentially expressed ubiquitin ligase–encoding genes in DN. (D) Heat map of the intersection of differentially expressed genes of ubiquitin ligases in DN. (E) STRING database identified the interaction between NEDD4 and PTEN. (F) Interaction between NEDD4 and PTEN was detected by Co‐IP. MPC5 cells were stimulated with NG + pGPU6, HG + pGPU6, or HG + pGPU6 − shMTDH for 24 h. mAb‐PTEN was used for immunoprecipitation, with rabbit IgG pAb as the control antibody. Western blotting was performed using mAb‐PTEN or mAb‐NEDD4. (G) Correlation analysis scatter plot of NEDD4 and PTEN in DN. (H, I) (H) Representative Western blot and (I) quantitative data on NEDD4 protein levels in four groups.  ^∗^ *p* < 0.05 (*n* = 3).

Through the STRING database, we found that MTDH may interact with PTEN (Figure 4A). Western blot results showed that under HG conditions, the interaction between PTEN and MTDH in MPC5 cells increased compared with NG conditions (Figure 4B). Next, we downloaded the GSE96804 dataset from the GEO database. We used this dataset to compare glomeruli obtained from DN kidneys against those from the unaffected sections of kidneys excised during tumor nephrectomy. From this dataset, we obtained 13,289 differentially expressed genes and compared them with the known ubiquitin ligase–encoding genes obtained from the Genecards database. The Venn diagram results indicate that the intersection region contains 120 ubiquitin ligase genes that are abnormally expressed in DN (Figure 4C). These genes may participate in the development of DN by regulating PTEN degradation. Based on the Venn diagram, we created an intersection heat map to more intuitively compare the differences between different intersection areas (Figure 4D). Based on the results from the STRING database (Figure 4E) and intersection heat map, we selected the ubiquitin ligase E3 NEDD4, which has been reported to negatively regulate PTEN protein levels in prostate, lung, and bladder carcinomas via poly‐ubiquitination and proteolytic degradation.

To clarify whether NEDD4 participates in the ubiquitination and degradation of PTEN in DN, we performed coimmunoprecipitation (Co‐IP) experiments. Co‐IP experiments confirmed that the binding between PTEN and NEDD4 was enhanced under HG conditions. Furthermore, this binding was reduced after downregulation of MTDH expression (Figure 4F).

Next, we obtained the expression levels of NEDD4 and PTEN from the GSE96804 dataset and performed a correlation analysis. The scatter plot showed a negative trend between NEDD4 and PTEN expression, although this association did not reach statistical significance (Figure 4G). To further investigate the effect of MTDH on NEDD4, we downregulated MTDH expression in MPC5 cells that had been treated with HG. WB analysis revealed that HG treatment led to increased NEDD4 expression in MPC5 cells; notably, the elevated NEDD4 expression triggered by HG intervention was mitigated after MTDH was downregulated. This suggests that suppressing MTDH expression can reverse the HG‐induced increase of NEDD4 in MPC5 cells (Figure 4H,I). Therefore, we preliminarily consider that MTDH can mediate the ubiquitination and degradation of PTEN through NEDD4 in MPC5 cells treated with HG.

### 2.5. Ubiquitin‐Conjugating Enzyme UBE2N Upregulated in Podocytes Exposed to HG

**Figure 5:**
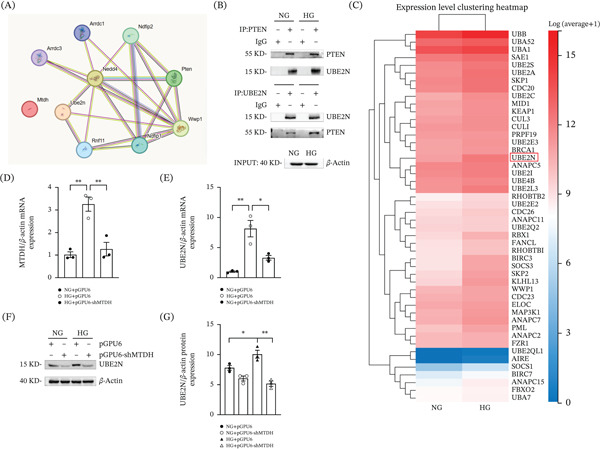
Ubiquitin‐conjugating enzyme UBE2N upregulated in podocytes exposed to HG. (A) STRING database identified the interaction between UBE2N, NEDD4, and PTEN. (B) Interaction between PTEN and UBE2N was detected by Co‐IP. MPC5 cells were stimulated with NG or HG for 48 h. mAb‐PTEN or mAb‐UBE2N was used for immunoprecipitation, with rabbit IgG pAb as the control antibody. Western blotting was performed using mAb‐PTEN or mAb‐UBE2N. (C) RNA sequencing identified 48 genes related to the ubiquitin‐mediated proteolysis pathway in MPC5 cells under HG conditions. (D, E) QPCR results showing MTDH and UBE2N expression in MPC5 cells across three groups.  ^∗^ *p* < 0.05,  ^∗∗^ *p* < 0.01 (*n* = 3). (F, G) (F) Representative Western blot results and (G) quantitative analyses of UBE2N protein levels in the four experimental groups are provided.  ^∗^ *p* < 0.05,  ^∗∗^ *p* < 0.01 (*n* = 3).

Through the STRING database, we found that ubiquitin‐conjugating enzyme UBE2N may interact with NEDD4 and PTEN (Figure 5A). Co‐IP experiments confirmed that the binding of PTEN to UBE2N was enhanced after HG conditions (Figure 5B). Combining the results of previous studies, we performed RNA sequencing in MPC5 cells under HG conditions. Through RNA sequencing, we identified 48 genes related to the ubiquitin‐mediated proteolysis that were upregulated in MPC5 cells under HG conditions, which included UBE2N (Figure 5C).

QPCR results demonstrated that HG stimulation of MPC5 cells led to increased mRNA transcription of UBE2N, which was significantly reduced following MTDH knockdown(Figure 5D,E). As shown by Western blot, the change in UBE2N protein level under the same intervention was consistent with the QPCR results (Figure 5F,G). This suggests that UBE2N may play a role downstream of MTDH under HG stimulation.

### 2.6. MTDH Promotes the Expression of Ubiquitin‐Conjugating Enzyme UBE2N, Leading to Increased Ubiquitination and Degradation of PTEN

**Figure 6:**
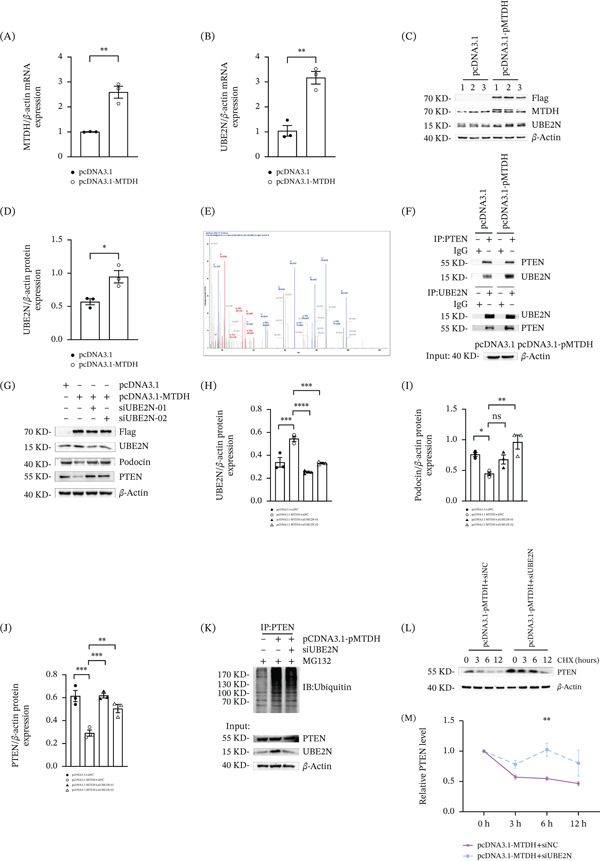
MTDH promotes the expression of ubiquitin‐conjugating enzyme UBE2N, leading to increased ubiquitination and degradation of PTEN. (A, B) QPCR results showing MTDH and UBE2N expression in MPC5 cells.  ^∗∗^ *p* < 0.01 versus pcDNA3.1 group (*n* = 3). (C, D) Western blot analysis showing UBE2N expression in MPC5 cells. (C) Representative Western blot results and (D) quantitative analyses of UBE2N expression are provided.  ^∗^ *p* < 0.05 versus pcDNA3.1 group (*n* = 3). (E) Mass spectrometry analysis of proteins binding to PTEN in podocytes overexpressing MTDH. UBE2N was detected. (F) Interaction between PTEN and UBE2N was detected by Co‐IP. MPC5 cells were stimulated with pcDNA3.1 or pcDNA3.1‐MTDH for 24 h. mAb‐PTEN or mAb‐UBE2N was used for immunoprecipitation, with rabbit IgG pAb as the control antibody. Western blotting was performed using mAb‐PTEN or mAb‐UBE2N. (G–J) (G) Representative Western blot and (H–J) quantitative data on UBE2N, podocin, and PTEN protein levels in four groups are presented. ns, no statistical difference;  ^∗^ *p* < 0.05,  ^∗∗^ *p* < 0.01,  ^∗∗∗^ *p* < 0.001,  ^∗∗∗∗^ *p* < 0.0001 (*n* = 3). (K) Coimmunoprecipitation demonstrating that PTEN binding to ubiquitin in MPC5 cells across three groups. (L, M) (L) Western blot analysis showing PTEN protein levels in indicated groups. MPC5 cells were transfected with pcDNA3.1‐MTDH and either siNC or siUBE2N for 24 h, then treated with CHX (40 *μ*M) and collected at the indicated time points for immunoblotting of PTEN and *β*‐actin. (M) Quantification of PTEN levels relative to *β*‐actin expression is shown.  ^∗∗^ *p* < 0.01 versus pcDNA3.1 − MTDH + siNC group.

To investigate whether there are expression changes in UBE2N in MPC5 cells overexpressing MTDH, we found that overexpression of MTDH directly upregulated the expression of UBE2N in MPC5 cells at both the mRNA and protein levels (Figure 6 A–D).

Next, mass spectrometry analysis indicated that there is an interaction between PTEN and UBE2N in MPC5 cells (Figure 6E). Co‐IP experiments confirmed that the binding of PTEN to UBE2N was enhanced after MTDH overexpression (Figure 6F). To further investigate the role of UBE2N in MTDH‐induced ubiquitination and degradation of PTEN, we used small interfering RNA (siRNA) targeting UBE2N (siUBE2N) to inhibit its expression. As shown in Figure 6G–J, downregulation of UBE2N in MPC5 cells reversed the decrease in PTEN and podocin expression caused by MTDH overexpression. To determine whether UBE2N is involved in the regulation of MTDH on PTEN through the ubiquitin–proteasome degradation mechanism, we further detected the ubiquitination level and protein degradation rate of PTEN following MTDH overexpression combined with UBE2N knockdown. As shown in Figure 6K–M, inhibition of UBE2N remarkably reduced MTDH‐induced ubiquitination of PTEN and delayed its degradation.

Our findings imply that MTDH contributes to the upregulation of the ubiquitin‐conjugating enzyme UBE2N, which in turn facilitates the ubiquitination and degradation of PTEN, ultimately leading to cytoskeleton rearrangement in podocytes.

## 3. Discussion

Glomerular podocyte injury is a well‐established contributor to the development of DN. The dense cytoskeletal network of FPs, which contains actin filaments, is connected to both the SD and GBM‐anchoring proteins (e.g., *α*3*β*1 integrins) [4]. Disruption of this network leads to FP effacement, where the FPs retract and the SD′s integrity is compromised, resulting in proteinuria [20]. Thus, cytoskeletal remodeling plays a pivotal role in driving the progression of DN.

MTDH, originally identified as an oncogene involved in tumor cell proliferation, migration, and apoptosis, is highly expressed in various malignancies, including breast, colorectal, and lung cancers [21, 22]. Our results reveal that MTDH levels were significantly elevated in glomerular podocytes from DN patients and db/db mice, suggesting a critical link between MTDH and podocyte injury in DN. Although previous research has demonstrated that MTDH contributes to podocyte apoptosis and renal inflammation [13, 23], our study identifies for the first time that MTDH functions as a critical regulator of podocyte cytoskeletal remodeling under diabetic conditions.

PTEN has established roles in cell proliferation, apoptosis, and migration [24, 25]. Recently, PTEN has been recognized as a crucial regulator of cytoskeletal dynamics. For instance, studies using PTEN knockout fibroblasts revealed that PTEN deficiency leads to the upregulation of signaling pathways associated with cell migration, including Ras‐related C3 botulinum toxin Substrate 1 (Rac1) and cell division control protein 42 homolog (cdc42), leading to enhanced cell motility [26]. In DN, PTEN deficiency has been associated with podocyte cytoskeletal disruption [27]. However, the specific regulatory mechanisms involved remain unclear.

In this study, we observed that reducing MTDH expression in HG‐treated podocytes alleviated PTEN loss, implicating a regulatory relationship between MTDH and PTEN stability. The ubiquitin–proteasome system is a well‐documented pathway regulating PTEN protein levels, with ubiquitination serving as a critical mechanism controlling PTEN′s subcellular localization and degradation. For example, studies in glioblastoma have demonstrated that increased expression of E3 ubiquitin ligases targeting PTEN promotes its degradation, thereby facilitating tumor progression [28]. Our data suggest that enhanced ubiquitination and subsequent degradation of PTEN, driven by elevated MTDH under HG conditions, represent an important mechanism underlying podocyte cytoskeletal remodeling in DN.

Ubiquitin–ligase E3 NEDD4 has been reported to negatively regulate PTEN protein levels through poly‐ubiquitination and proteolysis in carcinomas of the prostate, lung, and bladder. We found that there is a negative correlation between the expression of NEDD4 and PTEN in DN. Furthermore, the increased expression of NEDD4 in DN can be alleviated after downregulation of MTDH. To further investigate the ubiquitin‐mediated degradation mechanism of PTEN in DN, we utilized STRING database analysis and performed RNA sequencing. Utilizing RNA sequencing, we identified multiple genes related to ubiquitin‐mediated proteolysis upregulated in podocytes exposed to HG, including ubiquitin‐binding enzyme UBE2N. Proteomic analysis further revealed an interaction between PTEN and UBE2N, confirmed by Co‐IP assays. This interaction was significantly enhanced by MTDH overexpression. Furthermore, qPCR and Western blot analyses validated the role of MTDH in mediating HG‐induced UBE2N upregulation. Specifically, MTDH overexpression elevated UBE2N mRNA and protein levels, whereas MTDH knockdown reduced HG‐induced UBE2N expression. Crucially, inhibiting UBE2N expression effectively reversed MTDH‐mediated PTEN ubiquitination and degradation in podocytes. In the ubiquitin–proteasome system, ubiquitin‐conjugating enzyme E2 and ubiquitin ligases E3 work together to precisely regulate the ubiquitination modification and degradation of substrate proteins. We propose a reasonable hypothesis that in HG/MTDH driven PTEN degradation, NEDD4 is likely to act as an E3 ligase, forming a functional complex with UBE2N,an E2 ubiquitin‐conjugating enzyme, to jointly . This model conforms to the classic ubiquitination cascade reaction mechanism, that is, E2 and E3 work together to achieve substrate specific recognition and ubiquitin chain transfer. Nevertheless, whether UBE2N functions as an essential partner for NEDD4 in this process awaits further experimental validation. Future studies are warranted to biochemically validate the formation of a functional NEDD4‐UBE2N complex and its precise role in PTEN ubiquitination degradation under diabetic conditions.

Collectively, our findings provide new insights into the role of MTDH in the pathogenesis of DN, expanding the understanding of its functional scope. Although previous research has primarily focused on the regulation of inflammation and apoptosis by MTDH, our findings suggest that MTDH may contribute to disease progression by modulating podocyte cytoskeletal remodeling. Preliminary evidence indicates that MTDH overexpression may be associated with reduced PTEN stability and may participate in podocyte injury in DN by promoting its ubiquitin‐mediated degradation. These findings offer new clues for understanding the pathological role of MTDH in DN.

However, the mechanistic exploration in this study remains somewhat limited. The use of an immortalized cell line remains a limitation compared with primary podocytes or in vivo models. Although MPC5 cells are a widely used and well‐characterized model for podocyte studies, findings should be interpreted with caution and validated in more physiologically relevant systems in future work. Current data support MTDH interfering with actin homeostasis by downregulating PTEN. However, this study does not completely rule out the possibility of MTDH having a direct regulatory effect on cytoskeletal proteins or other related pathways that are independent of PTEN. In addition, the current results mainly reveal that UBE2N may act as an intermediary factor between MTDH and PTEN, extending to the DN context a regulatory relationship between MTDH and PTEN that has been observed in other diseases. Nevertheless, the specific regulatory mechanisms underlying this axis in DN remain unclear. The complete pathway through which MTDH regulates UBE2N and NEDD4 to mediate PTEN degradation—particularly whether MTDH directly regulates the expression or function of UBE2N—requires further in‐depth validation. Future research could build upon these findings to experimentally clarify the mode of regulation of UBE2N and PTEN by MTDH and, through more comprehensive mechanistic investigations, systematically elucidate the specific role of this signaling axis in DN.

## 4. Material and Methods

### 4.1. Animal Model

Six‐week‐old diabetic db/db mice (strain: C57BL/KsJ, BKS.Cg‐Dock7m+/+Leprdb/J) and their age‐matched normal littermates (genotype: db/m; five mice per group) of both sexes were purchased from the Model Animal Research Center of Nanjing University, China. All experimental animals were maintained under standard housing conditions, including a temperature range of 22°C–26°C, relative humidity of 40%–70%, and a 12‐h light/12‐h dark circadian cycle. The animals were sacrificed at 29 weeks of age. The study design and all experimental procedures adhered to the National Institutes of Health guidelines and were approved by the Ethics Committee for the experimental use of animals at Southern Medical University, Guangzhou, China.

### 4.2. Cell Culture and Treatment

Conditionally immortalized mouse podocytes (MPC5) were cultured as previously described [27, 28]. Briefly, undifferentiated MPC5 cells were maintained under permissive conditions at 33°C with 5% CO_2_ in RPMI 1640 medium (Gibco, United States) containing 10% fetal bovine serum (FBS; Gibco, United States), 100‐U/mL penicillin, 100‐*μ*g/mL streptomycin (Gibco BRL), and 50‐IU/mL recombinant murine IFN‐*γ* (Peprotech, United States). To induce differentiation, cells were transferred to 37°C (nonpermissive temperature) and cultured in RPMI 1640 medium containing 5% FBS, without IFN‐*γ*. Cells were treated with 5.3‐mM glucose (NG group), 5.3 − mM glucose + 25 − mM mannitol (M group), or 30‐mM glucose (HG group) for 24 or 48 h, as indicated. In some experiments, MPC5 cells were transiently transfected with plasmids or siRNA to regulate the expression of MTDH, PTEN, or UBE2N. To assess PTEN ubiquitination and degradation, cells were treated with MG132 (10 *μ*M) or CHX (40 *μ*M) for specific time periods. After treatment, cells were collected and subjected to various analyses.

### 4.3. Transient Transfection

MTDH or PTEN sequence was cloned into a pcDNA3.1 vector to construct a plasmid overexpressing MTDH (pcDNA3.1‐MTDH) or PTEN (pcDNA3.1‐PTEN). shRNA targeting MTDH or PTEN was cloned into a pGPU6 vector to knock down MTDH (pGPU6‐shMTDH) or PTEN (pGPU6‐shPTEN). The empty vector served as a negative control. All plasmids were provided by GenePharma. siUBE2N and its negative control were purchased from Ribobio. MPC5 cells were seeded into six‐well plates and, upon reaching 70% confluence, transfected using Lipofectamine 2000 (11668027; Invitrogen, Massachusetts, United States) along with the appropriate volume of plasmid. After 6 h, the transfection reagents were removed, and fresh complete medium supplemented with 5% FBS was added. The cells were then cultured for an additional 24 h before harvest.

### 4.4. The Transwell Invasion Assay

Twenty four hours posttransfection, the Transwell invasion experiment was conducted utilizing a modified Boyden chamber equipped with 8‐*μ*m pore‐size filters. MPC5 cells subjected to various treatment conditions were seeded into the upper compartment of the chamber, whereas the lower compartment was filled with cell culture medium. The chambers were then incubated for 24 h at 37°C in an incubator with a gas environment of 5% CO_2_ and 95% relative humidity. Following the incubation period, cells that had successfully migrated through the Matrigel‐coated membrane were fixed and stained to facilitate subsequent analytical procedures [29].

### 4.5. Real‐Time RT‐PCR

Total RNA was extracted from MPC5 cells using TRIzol reagent (Accurate Biology, China). mRNA levels were measured using a commercial kit (Accurate Biology, China) following the manufacturer′s instructions. Relative mRNA expression levels were determined using the comparative *Δ* *Δ*Ct method, as described previously [30].

### 4.6. RNA Sequencing and Bioinformatics Analysis

MPC5 cells were assigned to two groups (NG; HG), with three biological replicates included in each group. Total RNA was isolated using TRIzol Reagent (Life Technologies). RNA sequencing was carried out by the Beijing Genomics Institute, and the resulting data were analyzed via a bioinformatics platform. For differential expression analysis between the two groups, the DESeq2 tool was employed. The Benjamini and Hochberg method was used to calculate adjusted *p* values, which helped control the false discovery rate (FDR). Genes were defined as differentially expressed if they met two criteria: an adjusted *p* value < 0.01 and a fold change ≥ 2.

### 4.7. Western Blot Analysis

Protein specimens were prepared with radioimmunoprecipitation assay (RIPA) buffer, and their concentrations were quantified using a BCA kit (Thermo Fisher Scientific, United States). An equal load of protein (20 *μ*g per sample) was resolved on 12% sodium dodecyl sulfate–polyacrylamide gels (SDS‐PAGEs) and subsequently transferred onto polyvinylidene fluoride (PVDF) membranes. After being blocked with 5% skimmed milk in TBST for 1 h, the membranes were subjected to overnight incubation at 4°C with primary antibodies specific to MTDH (ab124789; Abcam), PTEN (9188S; Cell Signaling Technologies), nephrin (ab216341; Abcam), podocin (ab183703; Abcam), synaptopodin (sc‐515842; Santa Cruz), WT1 (ab89901; Abcam), FLAG (F1804; Sigma), ubiquitin (10201‐20AP; Proteintech), UBE2N (10243‐1‐AP; Proteintech), NEDD4 (21698‐1‐AP; Proteintech), and *β*‐actin (E021020‐01; EarthOx). Thereafter, the membranes were incubated with corresponding HRP‐conjugated secondary antibodies (E030110: Goat Anti‐Mouse IgG (*H* + *L*); E030120: Goat Anti‐Rabbit IgG (*H* + *L*); EarthOx) for 1 h under room temperature conditions. The intensity of protein bands was measured following the protocol described in prior studies.

### 4.8. Co‐IP

The interactions of PTEN or UBE2N with other proteins in MPC5 cells were assessed by Co‐IP. After treatment, cell lysates were immunoprecipitated overnight at 4°C using either anti‐PTEN (9188S; Cell Signaling Technologies) or anti‐UBE2N (10243‐1‐AP, Proteintech) antibodies, along with protein A/G plus agarose (sc‐2003; Santa Cruz Biotechnology). The immunoprecipitated complexes were washed three times with lysis buffer, boiled for 5 min in SDS sample buffer, and analyzed by immunoblotting.

### 4.9. LC‐MS/MS

Total cell lysates derived from MTDH‐overexpressing cells were subjected to affinity purification. The eluted purified protein complexes were resolved by SDS‐PAGE electrophoresis and subsequently visualized via silver staining. Protein bands of interest were excised from the gel, and shotgun proteomic analysis of these excised bands was performed by GeneChem.

### 4.10. Histology and Immunohistochemistry

Paraffin‐embedded sections prepared for immunohistochemical staining were first dewaxed and rehydrated. This was followed by antigen retrieval and inactivation of endogenous peroxidase activity. After blocking with 5% goat serum for 1 h at room temperature, the sections were incubated with primary antibodies at 4°C overnight. The primary antibodies used included anti‐MTDH (ab124789; Abcam) and anti‐PTEN (9188S; Cell Signaling Technologies). For signal detection, horseradish peroxidase–conjugated Goat Anti‐Rabbit or Anti‐Mouse secondary antibodies were applied, and the procedure was carried out in accordance with the manufacturer′s instructions (GK500705; Gene Tech).

Glomerular staining was quantified employing a histological scoring (*H*‐score) system, which integrates both the intensity of immunostaining and the proportion of positively stained cells. Immunostaining intensity was graded into four categories: *strong* (3+), *moderate* (2+), *weak* (1+), and *negative* (0). For each intensity grade, the percentage of podocytes exhibiting the corresponding staining was estimated and rounded to the nearest 10%. The *H*‐score was computed using the following formula: *H* − score = (0 × percentage of immunonegative cells) + (1 × percentage of weakly stained cells) + (2×percentage of moderately stained cells) + (3×percentage of strongly stained cells). The highest achievable *H*‐score was 300, which corresponds to 100% of cells displaying strong immunostaining. This scoring method enabled improved stratification of staining intensity across different samples, facilitating the distinction between high‐intensity and low‐intensity staining patterns. Moreover, this approach guarantees a quantitative and reproducible evaluation of protein expression levels in podocytes.

### 4.11. Phalloidin Staining

MPC5 cells exposed to distinct treatment conditions were fixed in 4% paraformaldehyde for 40 min at ambient temperature. Thereafter, cell permeabilization was performed using 0.5% Triton X‐100, with incubation at ambient temperature for 10 min. Subsequently, the MPC5 cells were incubated with 200 *μ*L of preprepared rhodamine phalloidin reagent (Cat. No. ab235137; Abcam) for 30 min at ambient temperature in the dark. Nuclei were counterstained using DAPI. Calculating the ratio of cells retaining distinct F‐actin fibers in different groups.

### 4.12. Human Tissue Specimens

Diagnostic renal biopsies and paracarcinoma normal kidney tissues were obtained from Zhujiang Hospital, Southern Medical University. All procedures involving human tissues received approval from the Zhujiang Hospital Institutional Ethics Committee (No. 2019‐KY‐068‐02).

### 4.13. Bioinformatics Analysis

We downloaded the GSE96804 dataset from the GEO database. We used this dataset to compare glomeruli obtained from DN kidneys against those from the unaffected sections of kidneys excised during tumor nephrectomy. From this dataset, we obtained differentially expressed genes and compared them with the known ubiquitin ligase–encoding genes obtained from the Genecards database. Obtain the intersection of ubiquitin ligase genes through a Venn diagram and draw a heat map. We obtained the expression levels of NEDD4 and PTEN from the GSE96804 dataset and performed a correlation analysis.

### 4.14. Statistical Analyses

All data are expressed as the mean ± SEM. Statistical analyses were performed using SPSS 19.0 (SPSS Inc., Chicago, Illinois, United States). Comparisons between two groups were performed using an unpaired Student’s t‐test. Comparisons among multiple groups were performed using one‐way analysis of variance followed by either the Student–Newman–Keuls test or Dunnett’s T3 test for post hoc comparisons, as appropriate. A *p* value < 0.05 was considered statistically significant.

**Nomenclature**
MTDH: metadherin
PTEN: phosphatase and tensin homolog
UBE2N: ubiquitin‐conjugating enzyme E2N
DN: diabetic nephropathy
ESRD: end‐stage renal disease
GBM: glomerular basement membrane
FPs: foot processes
SD: the slit diaphragm
AEG‐1: astrocyte elevated gene‐1
HIV: human immunodeficiency virus
TNF*α*
: tumor necrosis factor‐*α*
MPC5: mouse podocytes
NG: normal glucose
HG: high glucose
WT1: Wilms′ tumor Suppressor 1
CHX: cycloheximide
siRNA: small interfering RNA
shRNA: small hairpin RNA
IP: immunoprecipitation
Co‐IP: coimmunoprecipitation
LC‐MS/MS: liquid chromatography–tandem mass spectrometry
Rac1: Ras‐related C3 botulinum toxin Substrate 1
cdc42: cell division control Protein 42 homolog

## Author Contributions

**Zerong Zheng:** writing – original draft, visualization, validation, formal analysis, data curation. **Danping Tao:** writing – original draft, visualization, formal analysis, data curation. **Wenting Wu:** visualization, formal analysis, data curation. **Hui Zhang:** formal analysis, data curation. **Lingyu Shen:** validation, formal analysis, data curation. **Xiaohong Zheng:** formal analysis, data curation. **Yihao Long:** formal analysis. **Jinzhu Yang:** formal analysis. **Xiaowen Chen:** formal analysis, data curation. **Fenfen Peng:** writing – review and editing, formal analysis, data curation. **Haibo Long:**writing – original draft, visualization, project administration, methodology, formal analysis, data curation, conceptualization. **Congwei Luo:** writing – original draft, validation, supervision, project administration, methodology, conceptualization. **Zerong Zheng**, **Danping Tao**, and **Wenting Wu** have contributed to the work equally and should be regarded as cofirst authors.

## Funding

This work was supported by the National Natural Science Foundation of China (NSFC), (Grant Numbers 81900607, 82374198) (to Congwei Luo and Haibo Long), the Basic and Applied Basic Research Foundation of Guangdong Province, China, (Grant/Award Numbers 2019A1515011083) (to Congwei Luo), and the Science and Technology Action of Erdos High‐Tech Industrial Development Commission of Inner Mongolia (Grant/Award Number 2021XM08) (to Haibo Long).

## Ethics Statement

The human study was approved by the Medical Ethics Committee of Zhujiang Hospital, Southern Medical University (Document No. 2019‐KY‐068‐02). All patients provided written informed consent prior to participation. The animal study conformed to National Institutes of Health guidelines and was approved by the ethics committee for the experimental use of animals at Southern Medical University, Guangzhou, China (L2018136).

## Conflicts of Interest

The authors declare no conflicts of interest.

## Supporting information


**Supporting Information** Additional supporting information can be found online in the Supporting Information section. Figure S1: Validation of MPC5 podocyte differentiation and determination of high‐glucose treatment conditions. (A) Western blot analysis of synaptopodin and podocin expression in MPC5 cells cultured under proliferative (33°C, undifferentiated) and nonpermissive (37°C without IFN‐*γ*, differentiated) conditions. The results confirm the successful acquisition of a mature podocyte phenotype at 37°C, as indicated by the robust expression of differentiation markers.  ^∗^ *p* < 0.05 versus undifferentiated MPC5 cells (*n* = 3). (B) Western blot analysis showing MTDH and PTEN protein levels in vitro. MPC5 cells were stimulated with 5.3‐mM glucose (NG group), 20‐mM HG group, and 30‐mM HG group for 48 h. ns, no statistical difference;  ^∗^ *p* < 0.05,  ^∗∗^ *p* < 0.01 versus NG group.

## Data Availability

All data generated or analyzed during this study are included in this published article.
