# Supplementary figures and images for: Experimental Validation and Bioinformatics Analysis Elucidate the Role of MTDH‐Mediated PTEN Ubiquitination and Degradation in Podocyte Injury in Diabetic Kidney Disease
Source: Hum Mutat. 2026 May 13;2026:8914266. doi: 10.1155/humu/8914266 (PMC13171705; doi:10.1155/humu/8914266)

A

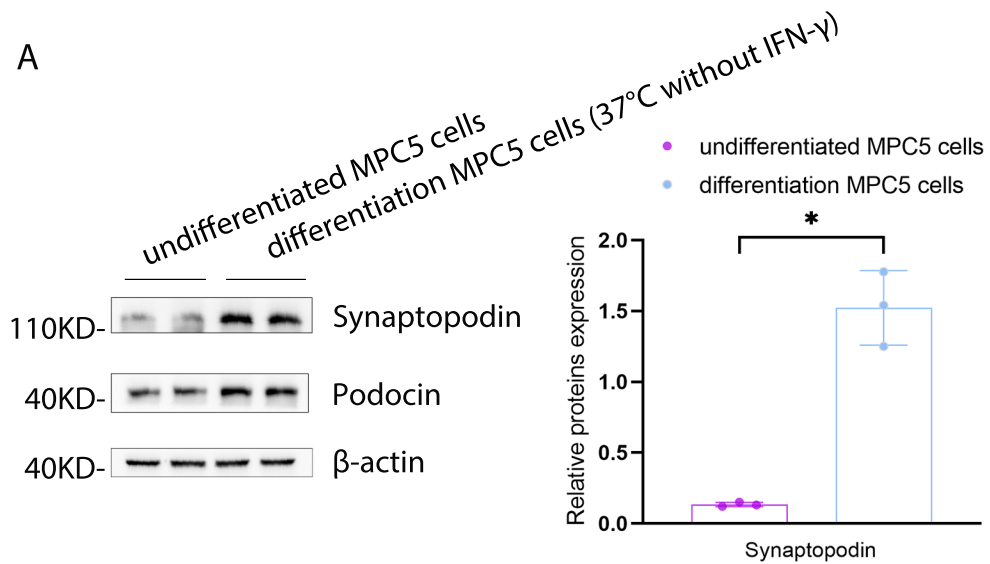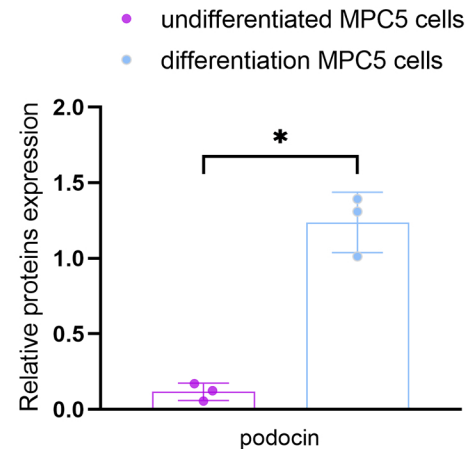

B

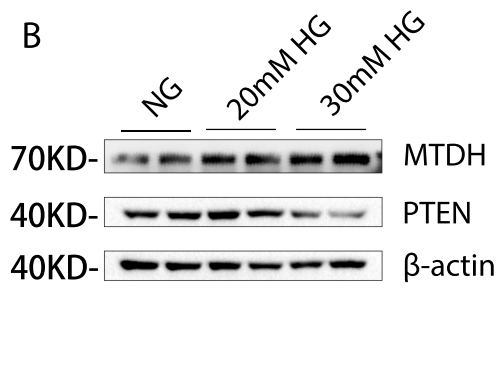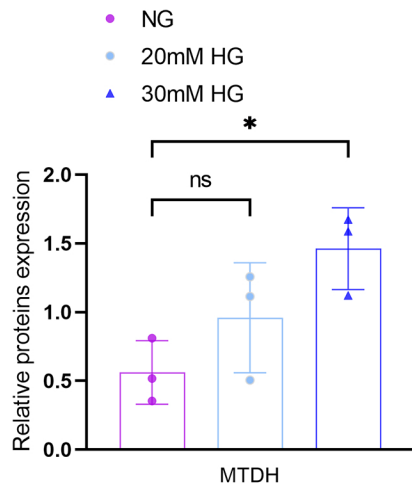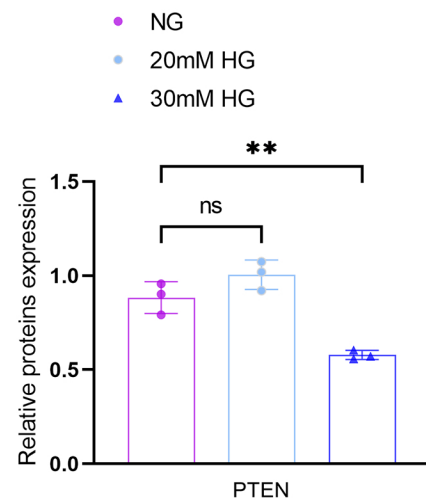

Supplement: Supplementary file 1 — Supporting Information Additional supporting information can be found online in the Supporting Information section. Figure S1: Validation of MPC5 podocyte differentiation and determination of high‐glucose treatment conditions. (A) Western blot analysis of synaptopodin and podocin expression in MPC5 cells cultured under proliferative (33°C, undifferentiated) and nonpermissive (37°C without IFN‐γ, differentiated) conditions. The results confirm the successful acquisition of a mature podocyte phenotype at 37°C, as indicated by the robust expression of differentiation markers. ∗ p < 0.05 versus undifferentiated MPC5 cells (n = 3). (B) Western blot analysis showing MTDH and PTEN protein levels in vitro. MPC5 cells were stimulated with 5.3‐mM glucose (NG group), 20‐mM HG group, and 30‐mM HG group for 48 h. ns, no statistical difference; ∗ p < 0.05, ∗∗ p < 0.01 versus NG group. [file HUMU-2026-8914266-s001.pdf]
